# Effects of *APOE* Genotype on Brain Proteomic Network and Cell Type Changes in Alzheimer's Disease

**DOI:** 10.3389/fnmol.2018.00454

**Published:** 2018-12-18

**Authors:** Jingting Dai, Erik C. B. Johnson, Eric B. Dammer, Duc M. Duong, Marla Gearing, James J. Lah, Allan I. Levey, Thomas S. Wingo, Nicholas T. Seyfried

**Affiliations:** ^1^Department of Neurology, Emory University School of Medicine, Atlanta, GA, United States; ^2^Department of Biochemistry, Emory University School of Medicine, Atlanta, GA, United States; ^3^Center for Neurodegenerative Diseases, Emory University School of Medicine, Atlanta, GA, United States; ^4^Department of Neurology, Second Xiangya Hospital, Central South University, Changsha, China; ^5^Department of Pathology and Laboratory Medicine, Emory University School of Medicine, Atlanta, GA, United States; ^6^Division of Neurology, Atlanta VA Medical Center, Decatur, GA, United States; ^7^Department of Human Genetics, Emory University School of Medicine,Atlanta, GA, United States

**Keywords:** Alzheimer's disease, proteomics, apolipoprotein E, inflammation, deconvolution

## Abstract

Polymorphic alleles in the apolipoprotein E (*APOE*) gene are the main genetic determinants of late-onset Alzheimer's disease (AD) risk. Individuals carrying the *APOE* E4 allele are at increased risk to develop AD compared to those carrying the more common E3 allele, whereas those carrying the E2 allele are at decreased risk for developing AD. How ApoE isoforms influence risk for AD remains unclear. To help fill this gap in knowledge, we performed a comparative unbiased mass spectrometry-based proteomic analysis of post-mortem brain cortical tissues from pathologically-defined AD or control cases of different *APOE* genotypes. Control cases (*n* = 10) were homozygous for the common E3 allele, whereas AD cases (*n* = 24) were equally distributed among E2/3, E3/3, and E4/4 genotypes. We used differential protein expression and co-expression analytical approaches to assess how changes in the brain proteome are related to *APOE* genotype. We observed similar levels of amyloid-β, but reduced levels of neurofibrillary tau, in E2/3 brains compared to E3/3 and E4/4 AD brains. Weighted co-expression network analysis revealed 33 modules of co-expressed proteins, 12 of which were significantly different by *APOE* genotype in AD. The modules that were significantly different by *APOE* genotype were associated with synaptic transmission and inflammation, among other biological processes. Deconvolution and analysis of brain cell type changes revealed that the E2 allele suppressed homeostatic and disease-associated cell type changes in astrocytes, microglia, oligodendroglia, and endothelia. The E2 allele-specific effect on brain cell type changes was validated in a separate cohort of 130 brains. Our systems-level proteomic analyses of AD brain reveal alterations in the brain proteome and brain cell types associated with allelic variants in *APOE*, and suggest further areas for investigation into the upstream mechanisms that drive ApoE-associated risk for AD.

## Introduction

Alzheimer's disease (AD) is the most common cause of dementia and affects millions of people worldwide (Prince et al., 2015), yet the biological basis of the disease remains poorly understood. Genetic analysis of late-onset AD has uncovered a number of single nucleotide polymorphisms (SNPs) that are associated with risk for the disease, but by far the strongest risk factor on a population level is genetic variation in the apolipoprotein E (*APOE*) gene (Strittmatter et al., 1993; Corder et al., 1994; Lambert et al., 2013). *APOE* is present in three common alleles within humans: ε2 (E2), ε3 (E3), and ε4 (E4). The E3 allele is the most common, with an allele frequency of 70–80%, while E2 and E4 are less common, with allele frequencies of 5–10% and 10–15%, respectively (Mahley and Rall, 2000). *APOE* E4 and E2 modify risk for AD. One E4 allele increases the relative odds to develop AD by 3.2, whereas two alleles increases the odds to 14.9. Consequently, E4 is present in 65–80% of all AD cases. The presence of an E2 allele lowers the odds to develop AD to 0.6 (Corder et al., 1994; Farrer et al., 1997). A number of hypotheses have been proposed to explain how the ApoE protein influences risk for AD, including effects on lipid metabolism, amyloid-β metabolism, mitochondrial function, cerebrovascular integrity, and inflammation (Huang, 2010; Verghese et al., 2011; Tai et al., 2016; Zhao et al., 2018). However, the mechanism(s) by which allelic variation in the *APOE* gene influences AD risk remains unclear.

Unbiased proteomic analysis of AD brain can yield insights into pathophysiological changes associated with the disease, and has yielded insights into alterations in individual proteins, groups of co-expressed proteins, biological processes, and cell type changes that are associated with AD (Seyfried et al., 2017). We recently described proteomic analyses of post-mortem brains from patients with AD, asymptomatic AD (AsymAD), and controls (Seyfried et al., 2017; Johnson et al., 2018). We found protein co-expression changes that correlated with both cognition and AD pathology, and were associated with AD risk loci identified by genome-wide association studies (GWAS). Many of the co-expression changes we identified were distinct from mRNA changes generated from a separate AD post-mortem brain cohort, suggesting that transcriptomic and proteomic analyses can generate both complementary and unique information. Many of these alterations were also strongly associated with glial biology, suggesting that glial activation is an important feature of AD progression and cognitive decline.

In this study, we used unbiased mass spectrometry-based proteomics to analyze the proteomes of 34 post-mortem control and AD brains of different *APOE* genotypes to better understand the brain proteomic changes associated with variation in the ApoE protein. We found that while there was no difference in amyloid-β burden among the *APOE* genotypes studied, E2/3 was associated with a lower tau burden compared to E3/3 and E4/4 AD cases. Protein co-expression analysis revealed that ApoE strongly correlated with modules involved in metabolism, peptidase activity, inflammation, and synaptic activity, and E2/3 was associated with less severe alterations in these protein coexpression modules. ApoE E2/3 also demonstrated resilience to “homeostatic” and “disease-associated” cell type changes across a number of different brain cell types—a finding that was replicated in a separate cohort of 130 brains. Collectively, these findings indicate that *APOE* variation impacts proteomic network and cell type-specific phenotypes in brain tissue.

## Materials and Methods

### Tissue Samples

Brain tissues used in the primary analysis were obtained from the autopsy collection of the Emory Alzheimer's Disease Research Center (ADRC) Brain Bank, and tissues used in the validation analysis were obtained from the Banner Sun Health Research Institute Brain Bank. Human postmortem tissues were acquired under proper Institutional Review Board (IRB) protocols with consent from family. In the Emory cohort, postmortem neuropathological evaluation of amyloid plaque distribution was performed according to the Consortium to Establish a Registry for Alzheimer's Disease (CERAD) criteria (Mirra et al., 1991) while extent of spread of neurofibrillary tangle pathology was assessed in accordance with the Braak staging system (Braak and Braak, 1991). Clinical and pathological information on all cases including disease status, *APOE* genotype, neuropathological criteria, age, sex, and post-mortem interval is provided in Supplementary Tables 1, 2. For the validation analysis, brain tissues were purchased from the Banner Sun Health Research Institute. A description of these tissues is provided in Supplementary Table 4 and Supplementary Data. Measures of AD pathology were derived in the Banner cohort as previously described (Beach et al., 2015).

### Tissue Homogenization and Protein Digestion

Each piece of tissue was individually weighed (~80 mg) and homogenized in 500 μL of urea lysis buffer (8 M urea, 100 mM NaHPO_4_, 10 mM Tris, pH 8.5), including 5 μL (100 × stock) HALT protease and phosphatase inhibitor cocktail (Thermo Scientific, Catalog #1861282), essentially as previously described (Seyfried et al., 2017). Homogenization was performed using a Bullet Blender (Next Advance) according to the manufacturer's protocols. Each tissue piece was added to urea lysis buffer in a 1.5 μL Rino tube (Next Advance) that contained 750 mg stainless steel beads (0.9–2 mm diameter) and homogenized twice for 3 min periods at 4°C. Homogenates were centrifuged for 5 min at 5,000 × *g* at 4°C, and the resulting supernatant transferred into 1.5 mL Eppendorf tubes and sonicated (Sonic Dismembrator, Fisher Scientific) three times (5 s, 30% amplitude, 12 s intervals between each sonication period), as previously reported (Seyfried et al., 2017; Umoh et al., 2018). Protein concentration was assessed using the bicinchoninic acid (BCA) method, and each homogenate was analyzed by SDS–PAGE to assess protein integrity. Samples were stored at 80°C. For protein digestion, brain protein homogenates (100 μg) were reduced with 1 mM dithiothreitol (DTT) at 25°C for 30 min and then alkylated using 5 mM iodoacetamide at 25°C for 30 min in the dark. Protein samples were digested with 1:100 (w/w) LysC at 25°C overnight, then diluted with 50 mM NH_4_HCO_3_ to a final concentration of 1 M urea and further digested with 1:50 (w/w) trypsin overnight at 25°C. The resulting peptides were desalted with a Sep-Pak C18 column (Waters) and then dried under vacuum using a SpeedVac concentrator (Labconco).

### Liquid Chromatography Coupled to Tandem Mass Spectrometry (LC-MS/MS)

Protein digests (2 μg) were resuspended in peptide loading buffer (0.1% trifluoroacetic acid). Peptide mixtures were separated on a self-packed C18 (1.9 μm, Dr. Maisch, Germany) fused silica column (50 cm × 75 μm internal diameter; New Objective, Woburn, MA) by an Ultimate 3,000 UHPLC (ThermoFisher Scientific, San Jose, CA) and monitored on an Orbitrap Fusion™ Tribrid™ mass spectrometer (ThermoFisher Scientific, San Jose, CA), as previously described (Umoh et al., 2018). Elution was performed over a 150 min gradient at a rate of 200 nL/min with buffer B ranging from 1 to 99% (buffer A: 0.1% formic acid in water, buffer B: 0.1% formic acid in acetonitrile). The mass spectrometer was programmed in a 3 s cycle during which the maximum number of data-dependent MS/MS scans were acquired. The MS scans (300–1,500 m/z range, 200,000 AGC, 50 ms maximum ion time) were collected at a resolution of 120,000 at m/z 200 in profile mode and the MS/MS spectra (1.5 m/z isolation width, 0.5 m/z offset, 30% HCD collision energy, 10,000 AGC target, 35 ms maximum ion time) were acquired using the ion trap in rapid mode. Dynamic exclusion was set to exclude previously sequenced precursor ions for 20 s within a 10 ppm window. Mass spectrometry analysis of the Banner cohort was performed on a Q-Exactive Plus (ThermoFisher) as previously described (Seyfried et al., 2017).

### Database Search and Quantification

Emory and Banner cohort RAW data files were analyzed separately using MaxQuant v1.6.0.1 and 1.5.3.30, respectively, with Thermo Foundation 3.0 and 2.0 for RAW file reading capability essentially as described (Seyfried et al., 2017) with slight modifications. The MaxQuant-integrated search engine Andromeda (Cox et al., 2011) was used to build and search a concatenated target-decoy Uniprot human reference database. The Uniprot database (downloaded 4/15/2015) included all Swissprot-curated (canonical) plus Trembl (unreviewed) sequences, totaling 90,411 FASTA sequence entries. Additional entries for unique peptides from ApoE E2 and E4 allelic gene products were included in the database. These were LLRDADDLQK**C**LAVYQAGAREGAER (R158C) for E2 and ELQAAQARLGADMEDV**R**GRLVQYR (C112R) for E4 alleles. Several Aβ-specific entries for peptides resulting from cleavages within the Aβ1-43 sequence were also included in the database. Protein methionine oxidation (+15.9949 Da), protein N-terminal acetylation (+42.0106 Da), and glutamine/asparagine deamidation (+0.9840 Da) were variable modifications (up to 5 allowed per peptide); cysteine was assigned a fixed carbamidomethyl modification (+57.0215 Da). Only tryptic peptides were considered, with up to 2 miscleavages per peptide, in the database search. A precursor mass tolerance of ±20 ppm was applied prior to mass accuracy calibration and ±4.5 ppm after internal MaxQuant calibration. Other search settings included a maximum peptide mass of 6,000 Da, a minimum peptide length of 6 residues, and 0.6 Da tolerance for ion trap HCD MS/MS scans (Emory acquired in the ion trap) or 0.05 Da for high-resolution MS/MS scans (Banner acquired in the Orbitrap). The false discovery rate (FDR) for peptide spectral matches, proteins, and site decoy fraction were all set to 1%. The label-free quantitation (LFQ) algorithm in MaxQuant was used for protein quantitation as previously described (Luber et al., 2010; Cox et al., 2014). To account for possible confounds in run time in the larger Banner cohort, a brain peptide standard, generated from pooled samples of homogenized brain, was included at different points in the run set to control for drift over time and highlight consistency in the protein measurements. Separate parameter groups were specified for Banner peptide precursor borrowing for LFQ within, but not across, four batches. To measure Tau MTBR and N-terminal signals, a separate search was performed using Proteome Discoverer 2.1 (ThermoFisher) with previously published parameters (Ping et al., 2018). All tau isoforms in the Uniprot human database were replicated as new “deltaMTBR” entries with the MTBR removed, and a separate entry for the MTBR encompassing residues 224–370 (of 441) in the Uniprot sequence (accession P10636-8) was added. LFQ intensity for amyloid-β was determined using the sum of MS1 chromatographic peak intensities for peptides HDSGYEVHHQK and LVFFAEDVGSNK, multiplied by the ratio of APP LFQ intensity divided by protein summed intensity, as previously described (Seyfried et al., 2017). Missing peptide-level measurements for these two peptides were imputed as the lowest non-missing value for the case status group. Protein abundance was determined by peptide precursor ion-intensity measurements across LC-MS runs using the label-free quantification (LFQ) algorithm in MaxQuant (Cox et al., 2014). All raw data and searched Maxquant files for Emory and Banner cohorts are deposited on Synapse (Emory Synapse ID 15623112, doi: 10.7303/syn15623112; Banner Synapse ID 7170616, doi: 10.7303/syn7170616).

### Protein Filtering and Missing Data Imputation

In total, 53,499 peptides mapping to 4,774 protein groups were identified among Emory case samples, and 99,130 peptides mapping to 5,711 protein groups were identified in the Banner cohort. However, one limitation of data-dependent label-free quantitative proteomics is missing quantitative measures, especially for low abundance proteins (Karpievitch et al., 2012; Seyfried et al., 2017). Thus, only those proteins quantified in >50% of samples were included in the data analysis. After filtering, and allowing up to < 50% (16 of 34) missing values across the LC-MS/MS runs, 4,382 unique proteins were identified and robustly quantified in the Emory cohort, and 3,710 unique proteins were identified and quantified in the Banner cohort. The missing protein LFQ values were imputed using the imputation algorithm of Perseus (Tyanova et al., 2016), where the Gaussian distribution of all log_2_-transformed non-missing protein quantifications has a mean, and 1.8 standard deviations less than this mean is considered as the mean of imputed missing values ± random noise distributed normally within 0.3 standard deviations, similar to what has been previously described (Seyfried et al., 2017).

### Outlier Removal and Regression

Outlier removal was performed prior to data analysis using an initial co-expression network connectivity-by-sample test, where samples with connectivity 3 standard deviations below the mean were removed, and the connectivity recalculated. This process was repeated until no more outliers could be detected, per Oldham's “SampleNetworks” v1.06 R script (Oldham et al., 2008) as previously described (Seyfried et al., 2017). Using this approach, 2 ApoE E4/E4 cases were removed from the 34 cases initially considered in the Emory cohort, and 9 of the 130 Banner cases considered were removed (Supplementary Table 1 and Supplementary Data).

Bootstrap regression on the remaining 32 (Emory) or 121 (Banner) -case LFQ intensity matrices was then performed, explicitly modeling case status category while removing covariation with age at death, gender, and post-mortem interval (PMI). Regression was followed by principal component analysis (PCA) of the expression data to confirm appropriate regression of selected traits, both in the “SampleNetworks” graphical output and via an in-house R script for PCA Spearman correlation to the traits for all non-outlier cases, pre- and post-regression. PCA visualized that the top five principal components had Spearman correlation rho < 0.3 with any of these three regressed covariates, and < 0.02 after regression.

### Co-expression Network Analysis

A weighted protein co-expression network analysis (WPCNA) was performed using the pre-processed protein abundance matrix by following previously described procedures for WGCNA (Seyfried et al., 2017), and using the WGCNA::blockwiseModules() function with the following settings: soft threshold power beta = 8.0, deepsplit = 4, minimum module size of 12, merge cut height of 0.07, signed network with partitioning about medioids (PAM) respecting the dendrogram and a reassignment threshold of *p* < 0.05. Specifically, the WGCNA:blockwiseModules() function calculates pair-wise biweight mid-correlations (bicor, a correlation metric robust to outliers) between each protein pair and transforms that matrix into a signed adjacency matrix (Langfelder and Horvath, 2012). The connection strength of components within this matrix is used to calculate a topological overlap matrix that represents measurements of protein expression pattern similarity across the set of samples in the cohort constructed on the pairwise correlations for all proteins within the network (Yip and Horvath, 2007). Hierarchical protein correlation clustering analysis by this approach is then conducted using 1-TOM, and initial module identifications are established using dynamic tree cutting; all these steps are implemented in the WGCNA::blockwiseModules() function (Langfelder and Horvath, 2008). Module eigenproteins, which represent the most representative abundance value for a module and which explain co-variance of all proteins within a module (Miller et al., 2013), were defined. Pearson correlations between each protein and each module eigenprotein were performed; this module membership measure is defined as kME.

### Differential Expression Analysis

Differentially enriched or depleted proteins (*p* < 0.05) were identified by ANOVA with Tukey's test comparing the four Emory clinical/genotype groups (*n* = 6 pairwise comparisons) and the five Banner clinical/genotype groups (*n* = 10 pairwise comparisons)(Umoh et al., 2018). Differential expression is presented as volcano plots, which were generated with the ggplot2 package in R and restricted to show only proteins arising from expression of one of the genes in the cell type-specific enrichment lists.

### Determination of Cell Type Marker Groups

Cell type-enriched markers for four cell types (neurons, oligodendrocytes, astrocytes, and microglia) were based on prior quantitative thresholding of LFQ abundance proteomic data from (Sharma et al., 2015), which resulted in comprehensive lists of proteins that distinguished these cell types in prior analyses we have performed (Seyfried et al., 2017). Here, we added to these four cell types a fifth cell type list for endothelia (derived from *Tie2*^+^ mouse brain-derived FACS-sorted cells) similarly quantitatively thresholded in-house, but where quantitative data originated from cell type-purified FPKM RNA-Seq data (Zhang et al., 2014b) quantified and compared to FPKM of all other purified brain cell types analyzed. Overlapping gene symbols found in the four other protein-derived cell type marker lists were removed from consideration as markers of those four cell types. This resulted in new culled exclusive gene symbol lists of 522 neuron, 411 oligodendroglia, 531 astrocyte, and 581 microglia cell type markers, and 1,149 endothelial markers, all from mouse. Gene symbols were converted to human symbols using the R biomaRt package (conversion against ensemble database performed on June 15, 2018), resulting in 460, 360, 468, 533, and 1079 respective human marker gene symbols for these five cell types.

### Digital Sorting Algorithm for Cell Type Proportion Analysis of Tissue Proteomes

Digital sorting of cell types was performed using the sample-wise unregressed abundances of proteins collapsed to a single measurement of maximum variance for each gene symbol in the abundance matrix overlapping with the above gene symbol lists, essentially as recently published (Johnson et al., 2018). Specifically, the DSA::EstimateWeight package and function, with method parameter set to “LM” (i.e., linear modeling), were used to calculate sample-wise five cell type weights or proportions of each sample (Zhong et al., 2013). Significance of changes across *APOE* genotype plus control/AD status-defined groups—independent of age, sex, and PMI effects—was then calculated as a Kruskal-Wallis *p* value using the R pf function applied to F-statistics, which were arrived at by summarization of the linear model of weights across samples after explicitly considering group, age, sex, and PMI.

### Synthetic Eigenprotein Calculations for Differentially Expressed Cell Type Protein Markers

After culling ANOVA-Tukey *p* value-based significant lists of proteins to only include cell type markers as defined above (repeated for each of the five cell types), proteins that were significantly changed in AD ApoE 3/3 vs. control 3/3 cases were divided into increasing and decreasing marker lists. Each sublist of protein isoforms identified by volcano calculations was then considered as a synthetic coexpression module definition and passed to the WGCNA::moduleEigengenes weighted eigengene calculator function, which calculated each synthetic eigenprotein using the full regressed protein abundance matrix. The same analysis was independently performed on sublists of proteins defined by the same protocol in the Banner cohort. *APOE* genotype plus control/AD status groups for case samples represented in the synthetic eigenproteins were then plotted with the R boxplot function. ANOVA was also performed for all Tukey pairwise comparisons across groups of cases for each eigenprotein.

### Other Bioinformatic Analyses

Gene Ontology (GO) functional annotation of modules was performed using GO-Elite 1.2.5 as previously published (Seyfried et al., 2017; Umoh et al., 2018), with a minimum of five genes per ontology, and a Fisher exact significance of *p* < 0.05, i.e., a Z-score >1.96. The background gene list for GO-Elite was all proteins considered for the network analysis, including proteins not assigned to a module (gray).

### SDS-PAGE and Western Blotting

Total brain homogenates (30 μg) in urea were mixed with Laemmli sample buffer and then resolved by SDS–PAGE using 10% or 4–12% Bolt Bis-Tris gels (Invitrogen) at 80 V for 10 min followed by 160 V for 35 min. Proteins were transferred onto nitrocellulose membranes (Invitrogen) using the iBlot 7 min dry transfer system (ThermoFisher Scientific). Membranes were blocked with TBS StartingBlock buffer (ThermoFisher Scientific) for 40 min at room temperature, and then probed overnight at 4°C with primary antibodies diluted in TBS StartingBlock buffer. Membranes were rinsed and incubated with secondary antibodies for 45 min at room temperature. Primary antibodies were Tau-5 (Invitrogen, MA5-12808, 1:1,000 dilution) and glyceraldehyde 3-phosphate dehydrogenase (GAPDH) (Abcam ab8245, 1:2,000), which was used as a loading control. The corresponding secondary antibodies were Alexa Fluor790 donkey anti-mouse IgG (H+L) (Invitrogen) (Invitrogen, A11371, 1:20,000) and Alexa Fluor680 goat anti-mouse IgG (H+L) (Invitrogen, A21058, 1:20,000). Membranes were imaged using an Odyssey Infrared Imaging system (LiCor Biosciences).

## Results

### ApoE 2/3 AD Brains Contain Similar Levels of Aβ but Less Tau Than E4/4 AD Brains

**Figure 1:**
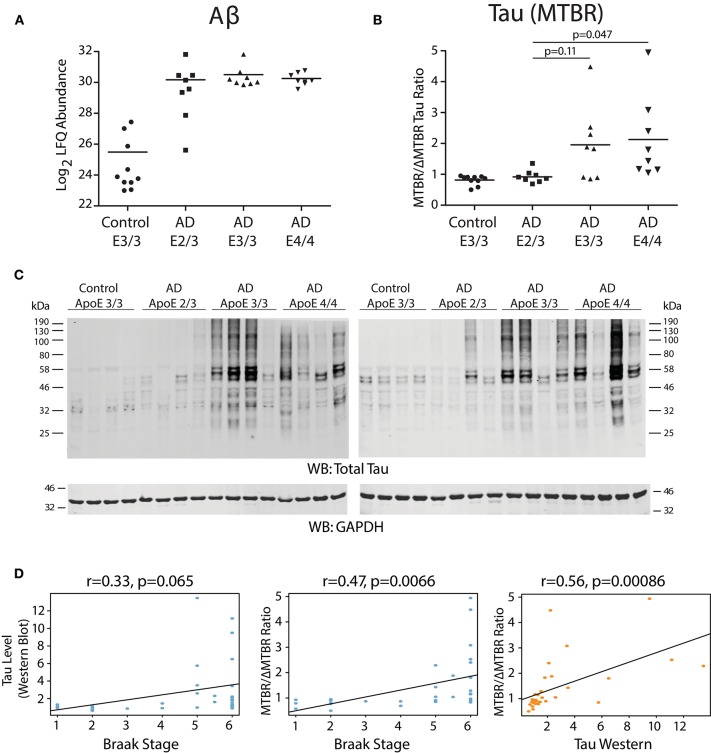
Effect of *APOE* Genotype on Amyloid-β and Tau Levels in AD Brain. **(A–D)** Amyloid-β (Aβ) levels in control and Alzheimer disease (AD) dorsolateral prefrontal cortex (DLPFC) brain region by *APOE* genotype, as measured by levels of the Aβ_6−16_ and Aβ_17−28_ peptides (see Methods) **(A)**. Levels of Aβ were not different among AD case groups by one-way ANOVA with Tukey's test. **(B)** Tau levels in control and AD DLPFC brain by *APOE* genotype as measured by the ratio of tau protein consisting of the microtubule-binding domain region (MTBR) only to the tau protein excluding the MTBR region (ΔMTBR). For illustration of tau protein level heterogeneity by protein region, see Supplementary Figure 1A. Levels of tau in AD E2/3 brains were significantly less than in AD E4/4 brains, were nearly significantly less than in AD E3/3 brains, and were not different from control E3/3 brains, by one-way ANOVA with Tukey's test. **(C)** Tau levels by western blotting for total tau using the Tau-5 antibody. Total tau levels by label-free quantification mass spectrometry (LFQ-MS) are shown in Supplementary Figure 1B. **(D)** Correlation between tau levels as measured by western blot densitometry to Braak stage (left panel), tau levels as measured by MTBR/ΔMTBR LFQ-MS to Braak stage (center panel), and tau levels as measured by MTBR/ΔMTBR LFQ-MS to western blot densitometry (right panel). Densitometry measurements are a sum of all tau species stained by the Tau-5 antibody. Correlations were performed using biweight midcorrelation (bicor).

We analyzed dorsolateral prefrontal cortex (DLPFC) from 34 Alzheimer's disease (AD) cases and controls with different ApoE genotypes from the Emory Brain Bank (Supplementary Table 1) by mass spectrometry-based proteomics. Our ApoE control and AD case groups were age-matched (Supplementary Table 2) to control for potential age-related brain proteome changes. Using “single-shot” label-free quantification (LFQ-MS), we were able to quantify the levels of 4,382 proteins across the experimental cohort. We first compared the levels of amyloid-β among ApoE genotypes based on quantification of the Aβ_6−16_ and Aβ_17−28_ peptides, which we have previously shown is a reliable marker for levels of amyloid-β and amyloid plaques as assessed by histopathology (Seyfried et al., 2017; Johnson et al., 2018). We did not observe a difference in the levels of amyloid-β by ApoE genotype in AD, although a few ApoE 2/3 carriers had lower amyloid-β levels compared to E3/3 and E4/4 (Figure 1A). We next assessed the levels of tau among ApoE genotypes. We noted that the levels of different tau peptide fragments vary considerably from controls in the AD brain (Supplementary Figure 1A), as has previously been shown (Sato et al., 2018). We therefore used peptides found within the tau microtubule binding region (MTBR) to quantify total tau levels, as this region of the protein aggregates into the neurofibrillary tangles found in AD and in other tauopathies (Serrano-Pozo et al., 2011; Lee and Leugers, 2012). We found that ApoE 2/3 carriers contained significantly less MTBR tau than ApoE 4/4 carriers (Figure 1B), suggesting a lower neurofibrillary tangle burden in E2 carriers. Total tau levels were also lower in ApoE 2/3 carriers by western blotting (Figure 1C), but were not significantly different by LFQ-MS given the aforementioned variability in levels of different tau fragments in the brain (Supplementary Figure 1B). Assessment of tau tangle burden by Braak stage correlated better with MTBR tau as measured by mass spectrometry than it did to total tau levels as measured by western blotting (Figure 1D), validating our approach to measurement of tau in the context of Alzheimer's disease pathology. Therefore, in patients who died with AD dementia, we observed similar levels of amyloid-β across ApoE 2/3, 3/3, and 4/4 genotypes in DLPFC, and lower levels of tau in E2/3 carriers compared to E4/4 carriers, with no difference in tau levels between E3/3 and E4/4 carriers.

### Protein Co-expression Network Analysis and Differential Protein Abundance by APOE Genotype

**Figure 2:**
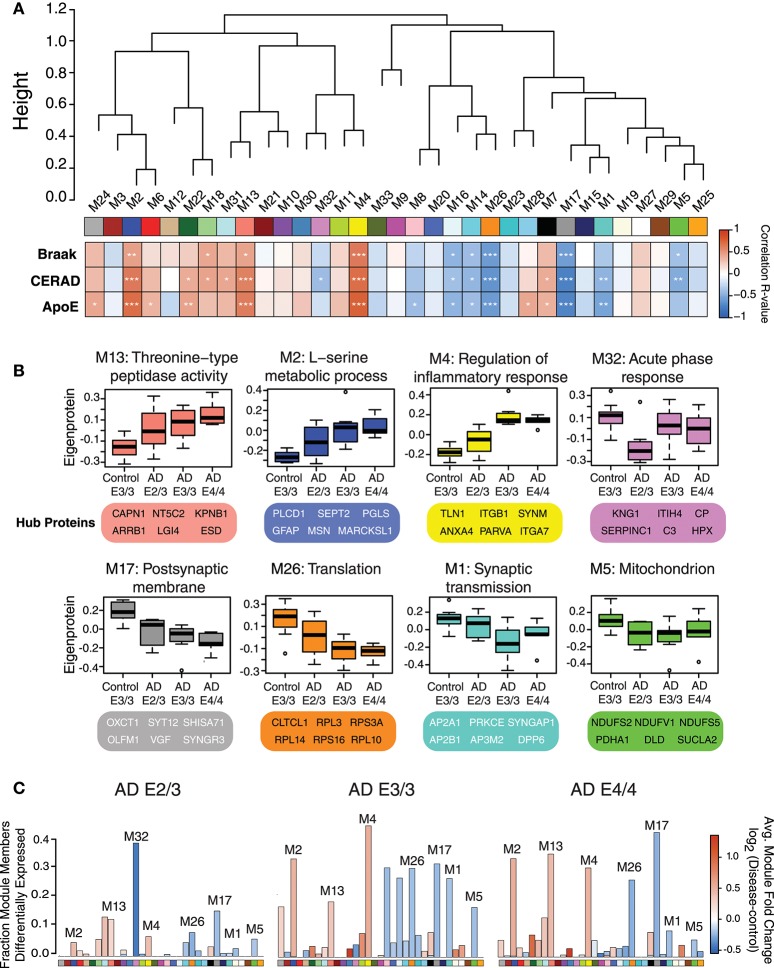
Protein Coexpression Network and Differential Abundance by *APOE* Genotype in AD**. (A–C)** Weighted Protein Correlational Network Analysis (WPCNA) was performed on 32 control and AD cases, and the resulting module eigenproteins were correlated to tau tangle burden (Braak stage), neuritic amyloid plaque burden (CERAD score), and *APOE* genotype using an ordinal scale where an E2 allele = −1, E3 = 0, and E4 = 1 (see main text) **(A)**. Strength of correlation to each trait is shown by heatmap, where red indicates positive correlation, and blue indicates negative correlation. Module-trait correlations were performed using biweight midcorrelation (bicor). ^*^p < 0.05, ^**^p < 0.01, ^***^p < 0.001. **(B)** Selected modules that showed significant trait correlations were analyzed by gene ontology (GO) analysis, with the resulting top GO term for each module listed as the module description. The most highly correlated proteins within each module (“hub proteins”) are listed below each module. Module eigenprotein values are plotted in control and AD *APOE* genotypes. **(C)** Differential protein abundance for each AD case group compared to control E3/3, by module. The height of the bars represents the fraction of module member proteins that had a difference in abundance compared to control. The bars are color coded by heatmap for average log2 difference in abundance, where red represents an increase in abundance in AD, and blue represents a decrease in abundance in AD.

We performed a weighted protein co-expression network analysis (WPCNA) on the ApoE cohort (Figure 2A) to identify groups of co-expressed proteins that correlated with AD endophenotypes. WPCNA identified 33 groups of co-expressed proteins (protein “modules”), many of which were significantly correlated with amyloid plaque or tau tangle burden. We also assessed whether each module was significantly correlated with ApoE status and ApoE-associated AD risk using an ordinal scale in which an E3 allele was equal to 0, E2 was equal to −1, and E4 was equal to 1 (therefore, an E4/E4 carrier would equal 2, while an E2/E3 carrier would equal −1). Modules that positively correlated to ApoE risk (E4>E3>E2) were M4, M2, M13, M22, M24, M28, M6, and M7 (Figure 2A, “ApoE” heatmap row). Modules that negatively correlated to ApoE risk (E2>E3>E4) were M17, M26, M1, M16, M8, and M14. Modules that correlated most strongly in a positive direction with ApoE included M2, M4, and M13, while those that correlated most strongly in a negative direction with ApoE were M1, M5, M17, M26, and M32. ApoE itself fell within the M4 module. To assess the biological processes associated with modules that correlated with ApoE-associated AD risk, we performed a gene ontology (GO) analysis for the most highly correlated modules (Figure 2B). Modules that correlated in a positive direction with ApoE were characterized by the top GO terms “threonine-type peptidase activity,” “L-serine metabolic process,” and “regulation of inflammatory response.” Those that correlated in a negative direction with ApoE were “acute phase response,” “postsynaptic membrane,” “translation,” “synaptic transmission,” and “mitochondrion.” We compared each AD ApoE variant to control E3/E3 to assess which modules contained the most differentially abundant proteins between disease and control for each variant (Figure 2C). Interestingly, module M32 contained a larger fraction of proteins that were decreased in ApoE 2/3 carriers relative to 3/3 and 4/4 carriers, and the magnitude of reduction was also larger, consistent with the module eigenprotein differences by *APOE* genotype. This was also the case for modules that correlated positively with ApoE variation, such as M2 and M4. Therefore, protein co-expression network analysis revealed modules that correlated with AD endophenotypes and *APOE* genotype, some of which had a substantial number of differentially abundant proteins between AD and control.

### ApoE Variants Affect Phenotypic Cell Type Changes in AD

**Figure 3:**
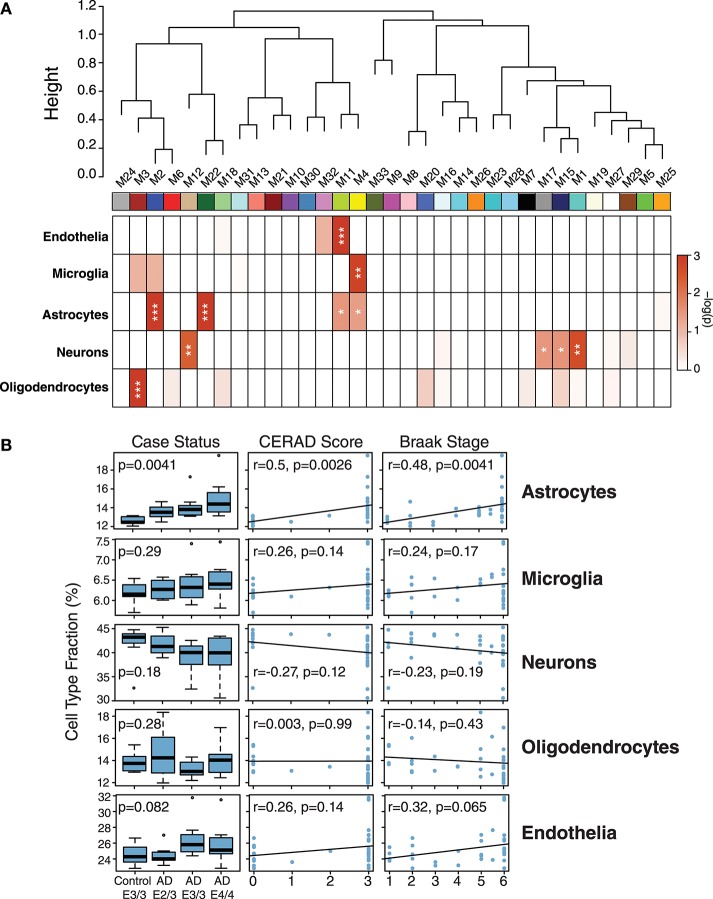
Network Module Cell Type Enrichment and Effect of *APOE* Genotype on Cell Type Changes in AD. **(A,B)** Cell type protein markers for microglia, astrocytes, neurons, and oligodendrocytes (Sharma et al., 2015)—and mRNA markers from endothelial cells (Zhang et al., 2014b)—from purified brain cell types were used to assess cell type enrichment for each network module by Fisher's exact test **(A)**. Significance of enrichment for a given cell type is shown by one-color heat map, with *p* values provided for selected cell type overlaps in Supplementary Table 3. *P* values were corrected by the Benjamini-Hochberg false discovery rate method. ^*^p < 0.05, ^**^p < 0.01, ^***^p < 0.001. **(B)** Cell type fraction estimation in control and AD cases by *APOE* genotype, and correlation of cell type fraction with amyloid plaque burden (CERAD score) and tau neurofibrillary tangle burden (Braak stage). Differences in cell type fraction among control and AD cases were assessed after one-way ANOVA. Correlations were performed using biweight midcorrelation (bicor).

**Figure 4:**
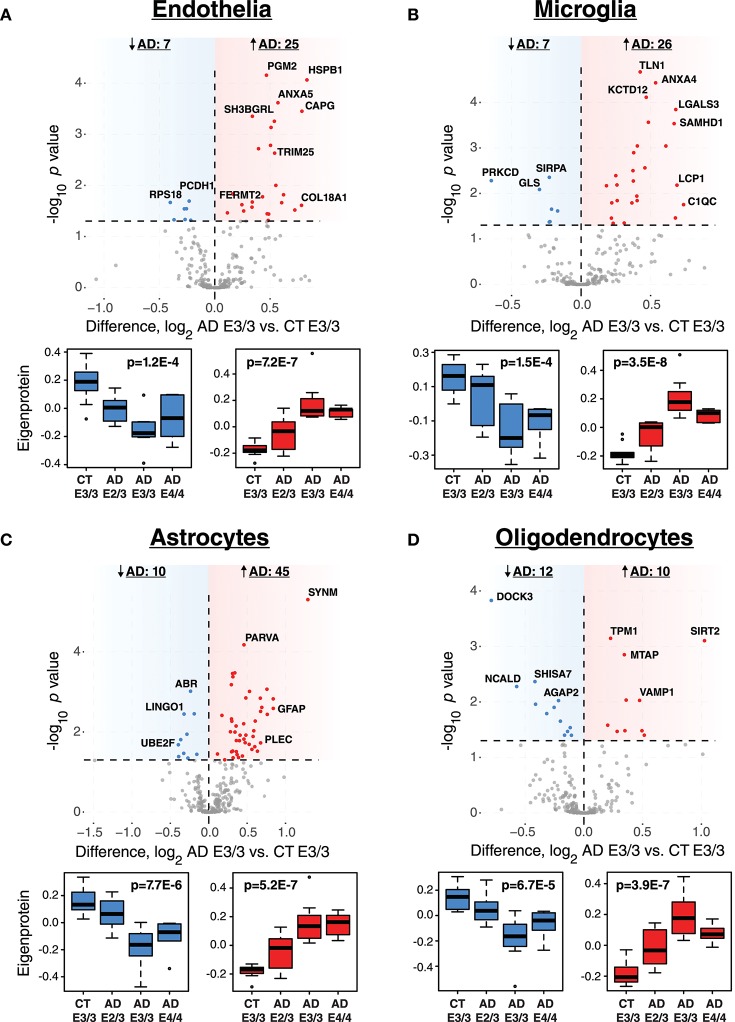
Differential Expression of Cell Type Markers in AD and Effects of *APOE* Genotype on Marker Expression. **(A–D)** Cell type markers for endothelia **(A)**, microglia **(B)**, astrocytes **(C)**, and oligodendrocytes **(D)** were analyzed for significant changes between control and AD on the ApoE 3/3 background. For those markers that were significantly increased (red) or decreased (blue) in AD E3/3 compared to control E3/3 brains (*p* < 0.05), a synthetic eigenprotein was generated for the significantly increased or decreased protein groups and analyzed for changes across *APOE* genotype by one-way ANOVA with Tukey's test. *P* values for one-way ANOVA are given in the box plots. Differences between AD E2/3 and control E3/3 were insignificant, while differences between AD E2/3 and AD E3/3 were significant, for decreased (or “homeostatic”) cell type marker eigenproteins for all cell types except endothelia (not significant for either comparison). Differences between AD E2/3 and AD E3/3 were significant for all increased (or “disease-associated”) cell type marker eigenproteins, while differences between AD E2/3 and control E3/3 were insignificant only for astrocytes (*p* = 0.06) and endothelia (*p* = 0.11). For a list of all *p* values after Tukey's test and a description of all markers, see Supplementary Data. CT, control; AD, Alzheimer's disease.

While ApoE is produced in the brain primarily by astrocytes (Boyles et al., 1985; Pitas et al., 1987), the cell type biological effects of ApoE that affect risk for AD are unclear. We performed a cell type enrichment analysis across the network using cell type protein marker lists derived from four purified brain cell types—neuron, astrocyte, microglia, and oligodendrocyte (Sharma et al., 2015)—in order to assess the cell type character of those modules that correlated with ApoE variation. In addition to these four brain cell types, we also included an endothelial protein marker list derived from RNAseq data of purified brain endothelial cells (Zhang et al., 2014b) to examine endothelial cell type enrichment, given the known effects of ApoE polymorphism on vascular biology and cerebrovascular disease (Bell et al., 2012; Schilling et al., 2013; Tai et al., 2016; Bouchareychas and Raffai, 2018). Analysis of cell type enrichment across the network revealed that modules M2 and M4, which correlated positively with *APOE* genotype, were strongly glial in nature, while modules M1 and M17 that correlated negatively with *APOE* genotype were neuronal in nature (Figure 3A, Supplementary Table 3). Interestingly, M32, which correlated negatively with ApoE, was largely endothelial, as well as its closely related module M11. However, M11 tended to correlate in an opposite direction with ApoE compared to M32. The anticorrelation with *APOE* genotype between two closely related endothelial modules prompted us to question whether there may be particular subpopulations within each cell type that are affected differently by allelic variation in *APOE*. To investigate this possibility, we first used a digital sorting algorithm (DSA) (Zhong et al., 2013) to estimate changes in bulk cell type abundance for the five different cell types by *APOE* genotype (Figure 3B), and correlated these changes in cell type abundance to amyloid plaque load and tau tangle burden. We observed that astrocytes increased in AD, and correlated with amyloid plaque and tau tangle burden. The fraction of astrocytes also correlated positively with ApoE status, suggesting that the astroglial response in AD is affected by ApoE. Interestingly, there was a trend toward a significant difference in fraction of endothelial cells, with elevated levels in AD E3/3 and E4/4 compared to control and AD E2/3. We then measured the differential expression of cell type specific proteins between AD and control for each of the five cell type protein marker lists. For all cell types except neurons, we found a significant number of cell type markers that were expressed in the opposite direction of what would be expected based on total cell type abundance measurements (Figure 4, Supplementary Figure 2). For future discussion and reference, we term groups of cell type markers that change in accordance with total cell type abundance “disease-associated,” while those that change in the opposite direction of total cell type abundance changes “homeostatic,” as has been previously suggested for different states of microglia function (Ransohoff and Perry, 2009; Liddelow et al., 2017; Rangaraju et al., 2018a,b). We assessed how ApoE variants might affect homeostatic and disease-associated cell subtype populations by measuring the levels of each disease-associated or homeostatic protein marker group (using a weighted principal component, or synthetic “eigenprotein,” for each marker group) among ApoE variants (Figure 4). We found that ApoE 2/3 suppressed both homeostatic and disease-associated cell subtype changes, while ApoE 4/4 had varied effects on phenotypic changes, compared to ApoE 3/3.

To validate that the observed effects of *APOE* genotype on disease-associated and homeostatic phenotypes were not specific to our particular cohort of AD cases, we analyzed a separate cohort of AD brains from the Banner Research Institute using the same methodology. This validation cohort consisted of 121 AD and control brains (Supplementary Table 4, Supplementary Data), and included ApoE 2/3 control brains to verify that the observed effects were specific to AD. In this separate cohort of AD brains we observed the same changes (significant or trend) in cell type subpopulations by ApoE status (Supplementary Figure 3), validating the findings in the Emory cohort, and confirmed that these effects were most pronounced in AD.

In summary, we found that certain protein co-expression modules were enriched for particular brain cell type markers, and that astrocytes were significantly increased in abundance in AD. Within each cell type studied—except for neurons—there was a significant fraction of cell type markers that changed in an opposite direction to bulk measurements, and both homeostatic and disease-associated marker changes were attenuated by ApoE 2/3.

## Discussion

In this study on the effects of *APOE* genotype on proteomic changes in Alzheimer's disease brain, we found that in patients who died with Alzheimer's disease dementia, those with *APOE* E2/3 genotype had 1) the same amount of amyloid-β burden in the brain but less neurofibrillary tau compared to E3/3 and E4/4 carriers; 2) fewer abnormal changes in protein coexpression modules that highly correlated with ApoE status and AD endophenotypes, including modules characterized by GO terms “regulation of inflammatory response,” “L-serine metabolic process,” and “threonine-type peptidase activity”; and 3) resilience to cell type changes associated with AD, including in homeostatic and disease-associated markers for each cell type.

The effects of *APOE* genotype on amyloid-β burden in the brain have been previously studied. ApoE has been found to regulate clearance of Aβ in an isoform-dependent fashion (E2>E3>E4) in animal models of AD and in humans without cognitive impairment (Castellano et al., 2011; Grothe et al., 2017). We did not observe a difference in Aβ burden among ApoE variants in our cohort, which may be due to analysis of brain at an end-stage of disease where Aβ neuritic plaque levels may have already plateaued, as suggested from post-mortem neuropathological analysis of Aβ plaque burden in E2 carriers (Serrano-Pozo et al., 2015). We observed a clear reduction of neurofibrillary tau, however, in E2/3 carriers compared to E3/3 and E4/4 carriers by LFQ-MS proteomics, consistent with previous neuropathological findings in a similar experimental cohort (Serrano-Pozo et al., 2015). We found that it was important to measure the MTBR domain of tau, rather than total tau, by mass spectrometry in order to obtain an accurate measurement of tau tangle burden. This is likely due to the fact that those regions of the tau protein that are not directly involved in the binding interactions between tau monomers to form tangles are removed and degraded in the brain, leading to low levels of these fragments and reduction of measured total tau levels if these fragments are included in abundance calculations (Sato et al., 2018). Therefore, peptide- and domain-level measurements for tau are an important consideration for measurement of this protein by standard mass spectrometry proteomic approaches.

Although we do not have cognitive data in our cohort to assess the relationship of ApoE status to cognitive function, prior studies have shown that E2 carriers show less severe cognitive deficits compared to E3 and E4 carriers (Serrano-Pozo et al., 2015), even when levels of pathological forms of tau and Aβ are similar (Berlau et al., 2009), suggesting that the protective effects of the E2 allele may be independent from its effects on Aβ and tau pathology. Our systems-level analysis suggests other potential mediators of the ApoE effect on cognitive decline. Protein co-expression modules characterized by GO terms “L-serine metabolic process,” “threonine-type peptidase activity,” “regulation of inflammatory response,” “translation,” “post-synaptic membrane,” and “synaptic transmission” were strongly associated with variation in ApoE. It is currently unknown the extent to which each of these protein modules is an upstream driver of the ApoE effect, but we find it notable that one of the modules most strongly correlated with ApoE was the inflammatory module M4. ApoE has been shown to regulate immune function in mouse models of AD and other diseases, and may be a key regulator of the brain's response to neurofibrillary tangle formation (Stoll and Bendszus, 2006; Shi et al., 2017). However, a direct neuronal effect of ApoE is also possible given “post-synaptic membrane” and “synaptic transmission” module changes. ApoE can be expressed in neurons under conditions of cellular stress (Boschert et al., 1999), and lead to tau phosphorylation as well as mitochondrial toxicity (Chang et al., 2005; Wang et al., 2018). These possibilities are not mutually exclusive, and require further study to define their potential contributions to AD pathology in humans.

While deconvolution of cell type abundance from bulk tissue measurements in AD brain has previously been performed (Li et al., 2018), a novel aspect of our study is that we assessed for phenotypic changes associated with each cell type analyzed—neurons, astrocytes, microglia, oligodendrocytes, and endothelia—as reflected by markers for each cell type that increase or decrease in AD. We term these “homeostatic” and “disease-associated” cell phenotypes, as previously suggested for microglia (Ransohoff and Perry, 2009; Liddelow et al., 2017; Rangaraju et al., 2018a,b) and, in a similar fashion, for astrocytes (Zamanian et al., 2012). Parsing phenotypic cell type changes in such a manner leads to additional insight into cell type changes in AD beyond bulk changes assessed by deconvolution. We found that the ApoE 2/3 variant suppressed almost all homeostatic and disease-associated AD cell type changes, while the E4/4 variant did not show increased severity of these phenotypes compared to E3/3. These observations were replicated in a separate cohort of brains, suggesting that they are not unique to our Emory cohort. One possibility for the minimal difference between E3/3 and E4/4 carriers in cellular phenotypes is the stage at which we analyzed the tissues, where at end-stage the differences in pathologies may have largely plateaued between these two variants, as suggested from the Aβ and MTBR tau measurements. The observed suppression of the disease-associated microglia phenotype by the E2 allele in our study is a corollary finding to the reported suppression of an aged microglia phenotype by E2 in post-mortem human brain, as well as the lack of an E4 effect on aged microglia (Olah et al., 2018). The suppression of homeostatic and disease-associated microglial changes by E2 further supports an ApoE effect on brain inflammatory response pathways.

Another novel aspect of our study is that, in addition to four other canonical brain cell types, we assessed for endothelial cell type enrichment and changes by ApoE status given the role of ApoE in vascular disease (Bell et al., 2012; Schilling et al., 2013; Tai et al., 2016; Bouchareychas and Raffai, 2018). Deconvolution of cell type showed that the E2 allele prevented an increase in endothelial cells in AD, and tended to suppress changes in homeostatic and disease-associated endothelia. The E2 effects on endothelia and oligodendrocytes are noteworthy given the robust observation that E2 carriers have a larger burden of white matter hyperintensities on MRI compared to E3 and E4 carriers (Schmidt et al., 1997; Lemmens et al., 2007; Raz et al., 2012; Schilling et al., 2013)—which are often considered to reflect vascular pathology and/or myelination changes—and have a higher risk for brain infarction and lobar intracranial hemorrhage compared to E3 carriers (Biffi et al., 2010, 2011; Schilling et al., 2013). This is despite the fact that E2 lowers total cholesterol and LDL (Sing and Davignon, 1985; Schmidt et al., 1997). There are two caveats to our endothelial cell type analysis. One is that mRNA rather than protein markers were used to define the endothelial cell type. The correlation between mRNA and protein levels can vary substantially, and is often of moderate strength (Zhang et al., 2014a; Seyfried et al., 2017; Olah et al., 2018). Therefore, use of an mRNA-derived marker list may have introduced some variability in our analysis of endothelial cells, and an endothelial marker list derived from protein measurements on purified endothelial cells would be a useful contribution to proteomic endothelial cell type analyses. The second caveat is that we enforced exclusion of markers to a particular cell type. In reality, such markers are rarely exclusively expressed in a given cell type, and this is especially true for endothelia and microglia given their common mesodermal embryonic origin (Zhang et al., 2014b). The protein co-expression network reflects this similarity in cell type markers between endothelia and microglia, where two highly related modules M4 and M11 were found to be strongly enriched in microglial (M4) and endothelial (M11) markers. In future analyses, it may be interesting to include a “micro/endo” phenotype for cell type deconvolution. Nevertheless, the relationship between E2 and endothelial and oligodendrocyte changes is intriguing, and deserves further study in the context of AD risk modification. A final caveat is that our primary cohort contained E3/3 only as the control group with which to compare E2/3, E3/3, and E4/4 effects. Our validation cohort contained an E2/3 control group that was very similar to E3/3 in the cell type marker analysis, suggesting that E2 effects are most evident in AD. However, future studies would benefit from exactly matched ApoE controls, even though these can be difficult to obtain due to the low population frequencies of the E2 and E4 alleles and the elevated AD risk imparted by E4/4.

In summary, our analysis of AD brain revealed proteomic and cell type changes influenced by *APOE* genotype, and suggests that ApoE may influence AD risk through a combination of effects on inflammation, metabolism, and cerebral vasculature, rather than through direct effects mediated by amyloid-β and tau.

## Data and Software Availability

Emory ApoE cohort RAW data, MaxQuant output, and analysis are deposited at the Sage Synapse website with doi: 10.7303/syn15623112, accessible via the URL https://www.synapse.org/#!Synapse:syn15623112. Banner RAW data and full MaxQuant output have also been deposited at the Synapse website with doi: 10.7303/syn7170616, accessible via the URL https://www.synapse.org/#!Synapse:syn7170616. Software is available upon request.

## Author Contributions

JD, ED, EJ, TW, NS, AL, and JL: conceptualization; DD, ED, JD, EJ, TW, and NS: methodology; JD and DD: investigation; ED, JD, EJ, and NS: formal analysis; EJ, JD, and ED: writing – original draft; EJ, JD, ED, DD, MG, TW, NS, JL, and AL: writing – review and editing; AL and NS: funding acquisition; MG: resources.

### Conflict of Interest Statement

The authors declare that the research was conducted in the absence of any commercial or financial relationships that could be construed as a potential conflict of interest.
