# Supplementary material for: Effects of APOE Genotype on Brain Proteomic Network and Cell Type Changes in Alzheimer's Disease
Source: Front Mol Neurosci. 2018 Dec 18;11:454. doi: 10.3389/fnmol.2018.00454 (PMC6305300; doi:10.3389/fnmol.2018.00454)
Supplement: Supplementary file 10 [file Data_Sheet_5.docx]

**Supplementary Table Legends**

**Supplementary Table 1. Emory Cohort Samples**. Samples with an asterisk (*) were not included in the protein co-expression network analysis because of their outlier status by network connectivity (see Methods). AD, Alzheimer’s disease; *APOE*, apolipoprotein E isoform genotype; CT, control; CERAD, Consortium to Establish a Registry for Alzheimer’s Disease amyloid-β plaque load score; Braak, Braak stage for tau tangle burden; PMI, post-mortem interval.

**Supplementary Table 2. Emory Case Characteristics by *APOE* Genotype**. Values shown are means ± SD. AD, Alzheimer’s disease; CERAD, Consortium to Establish a Registry for Alzheimer’s Disease amyloid-β plaque load score; Braak, Braak stage for tau tangle burden; PMI, post-mortem interval.

**Supplementary Table 3. Network Module Cell Type Enrichment *P* Values.** FET, Fisher’s exact test. *P* value for enrichment of endothelia markers in module M11 is highlighted in bold.

**Supplementary Table 4. Banner Case Characteristics by *APOE* Genotype**. Values shown are means ± SD from the final 121 case cohort. A description of the individual samples is provided in **Supplementary Data**. AD, Alzheimer’s disease; CERAD, Consortium to Establish a Registry for Alzheimer’s Disease amyloid-β plaque density score; Braak, Braak stage for tau tangle burden; PMI, post-mortem interval.

**Supplementary Figure Legends**

**Supplementary Figure 1. Quantification of Full-Length Tau Protein by Mass Spectrometry.** **(A, B)** Schematic of full-length tau protein containing the two N-terminal repeat domains (2N) and four microtubule-binding repeat (MTBR) domains (4R) **(A)**. Peptide fragments used for full-length protein quantification by label-free quantification mass spectrometry (LFQ-MS) are shown below according to the approximate region of the tau protein from which they are derived, and are color coded according to whether the peptide is increased (red) or decreased (blue) in Alzheimer’s disease vs. control. Levels of selected peptides by *APOE* genotype within and outside the MTBR are given as general examples. Tau schematic and peptide fragments are not drawn to scale. Three out of the 31 measured tau peptides did not map to the 441 amino acid tau isoform, and are therefore not shown. **(B)** Quantification of full-length tau by LFQ-MS across case groups in the Emory cohort. Differences in tau levels were not significant after one-way ANOVA.

**Supplementary Figure 2. Differential Expression of Neuronal Cell Type Markers in AD**. Neuronal cell type markers significantly increased (red) or decreased (blue) in AD vs. control on the ApoE 3/3 background are shown. For a list of all markers, see **Supplementary Data**. CT, control; AD, Alzheimer’s disease.

**Supplementary Figure 3. Effects of *APOE* Genotype on Cell Subtype Marker Expression in the Banner Cohort.** Eigenproteins for protein groups that were significantly increased (red, disease-associated) or decreased (blue, homeostatic) in AD compared to control on the ApoE 3/3 background were measured across *APOE* genotypes (CT E2/3 *n*=18, CT E3/3 *n*=59, AD E2/3 *n*=6, AD E3/3 *n*=30, AD E4/4 *n*=8) in the Banner cohort (see **Supplementary Data**). Eigenprotein differences were significant by one-way ANOVA for all groups. For a list of all post-hoc comparisons by Tukey’s test, see **Supplementary Data**. CT, control; AD, Alzheimer’s disease.

**
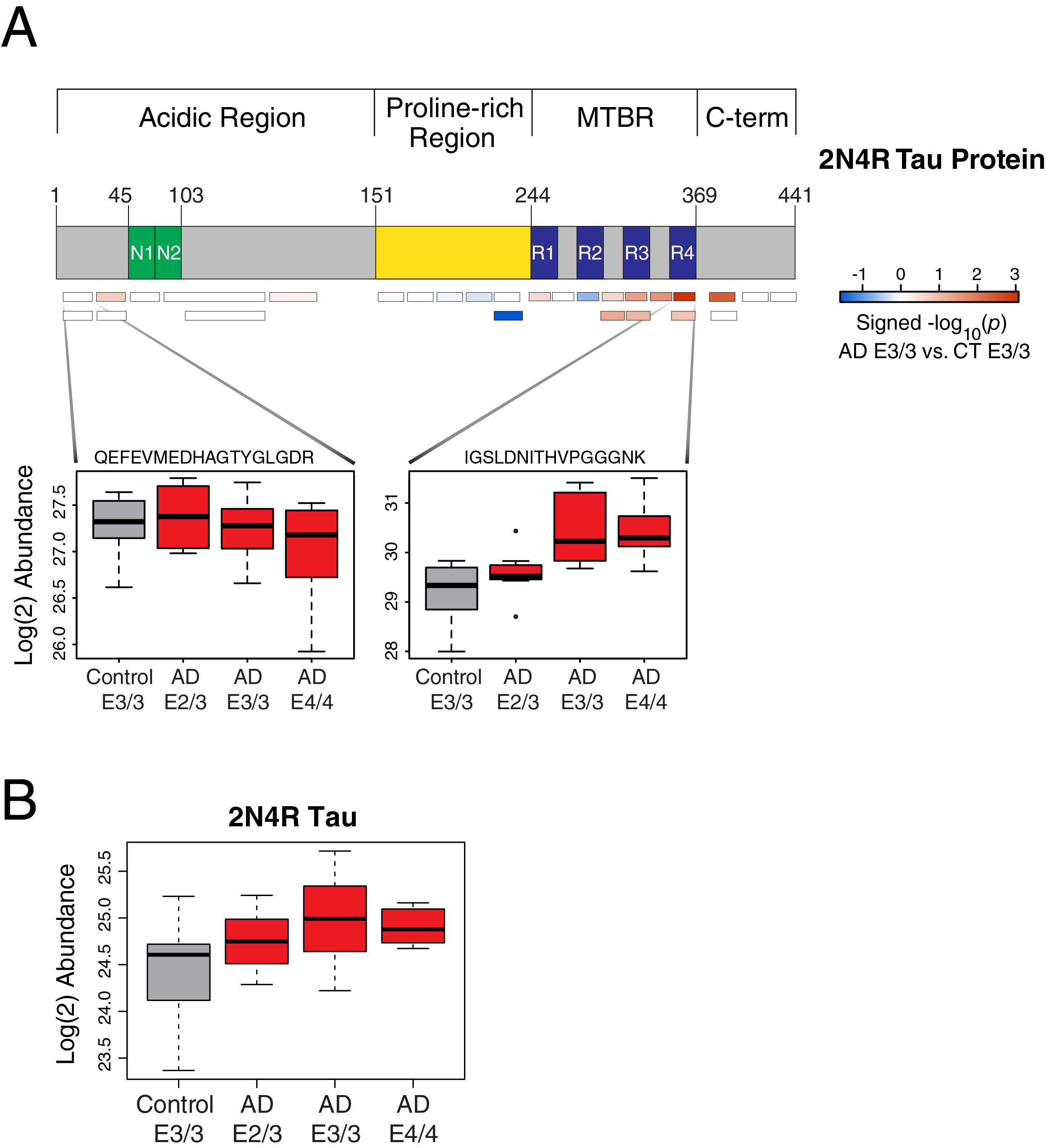
**

**Supplementary Figure 1**

**
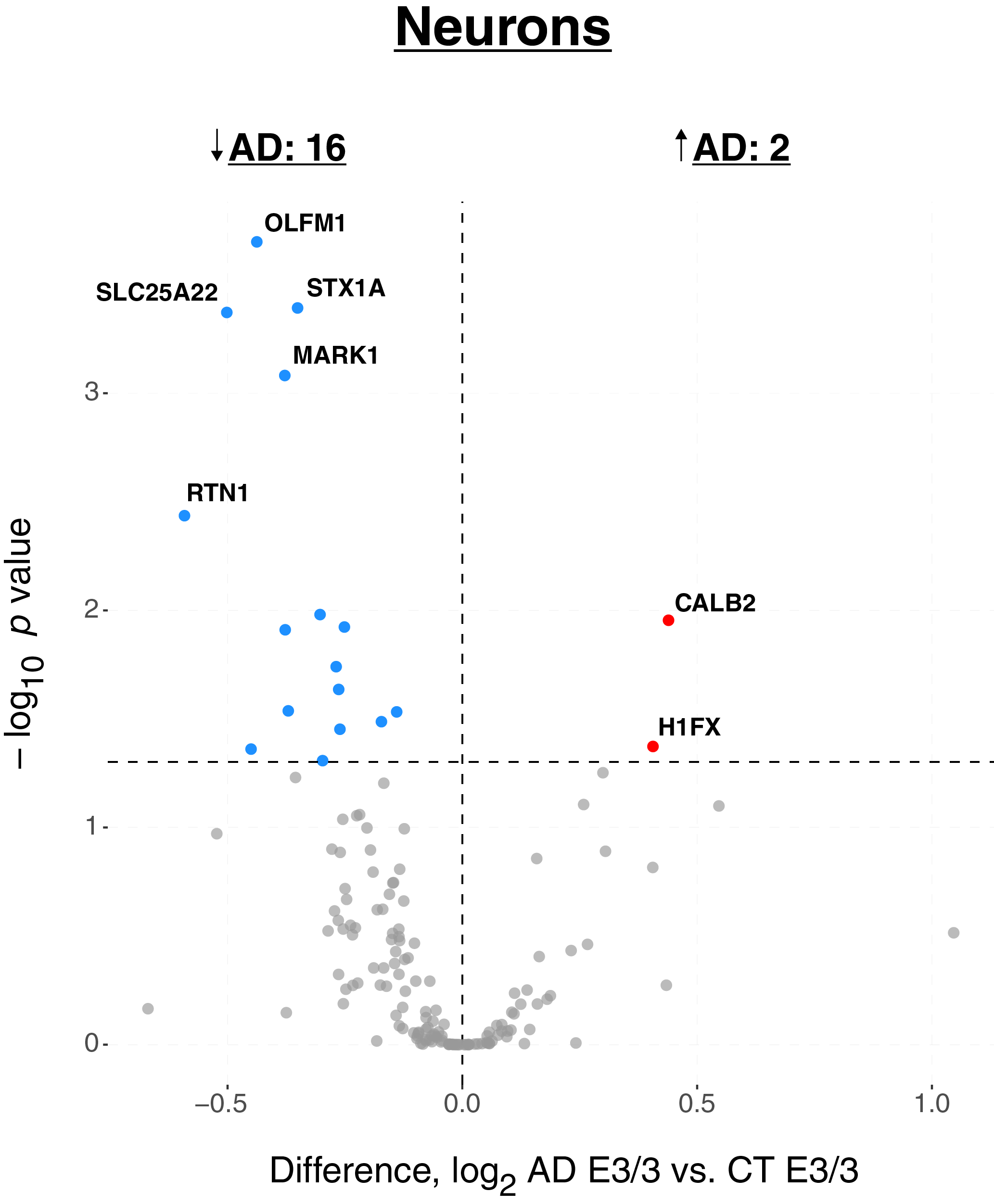
**

**Supplementary Figure 2**

**
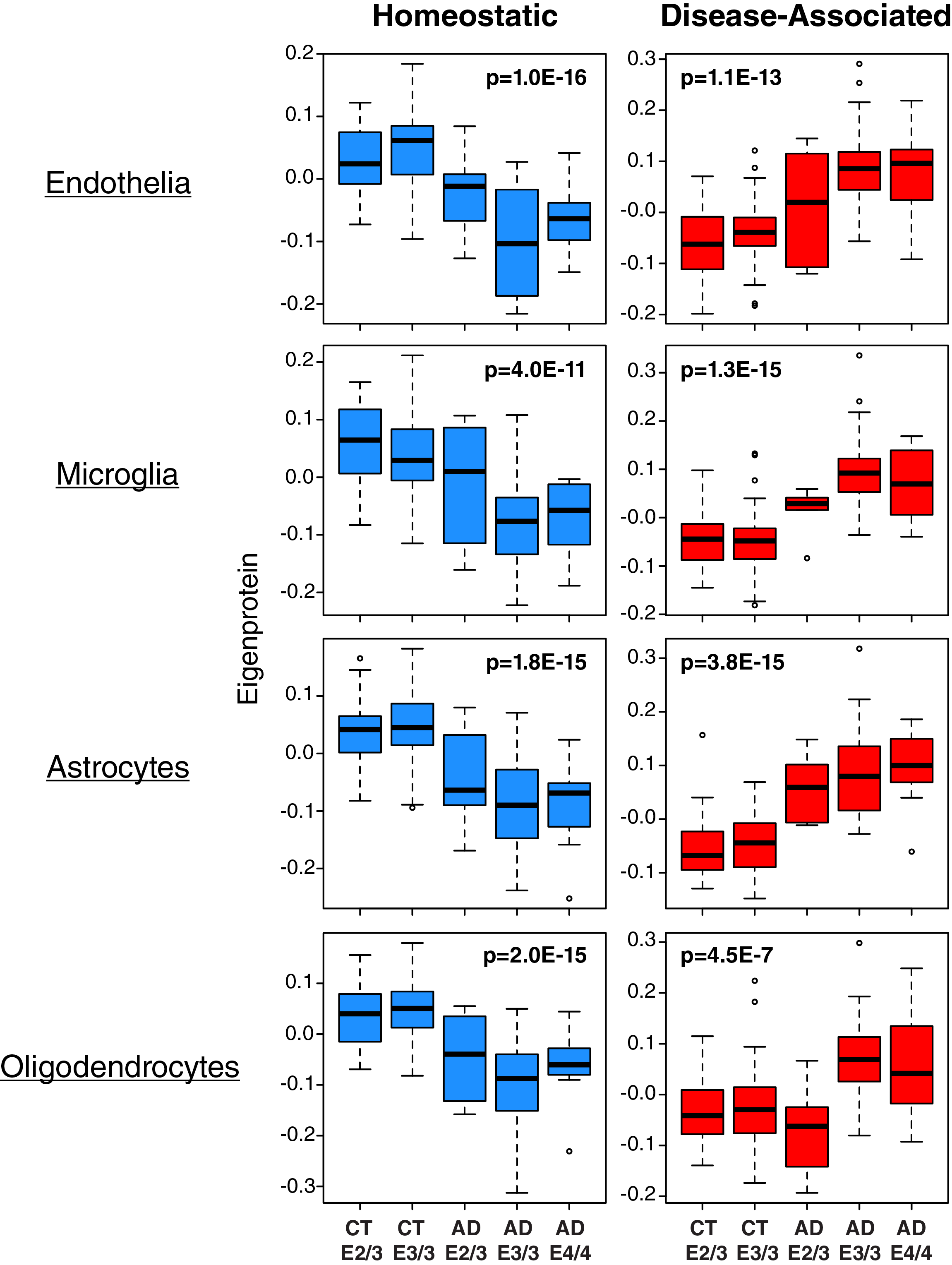
**

**Supplementary Figure 3**

| Case ID | *APOE* | CERAD | Braak | Age | Sex | PMI (h) |
| --- | --- | --- | --- | --- | --- | --- |
| CT1 | 3/3 | 0 | 1 | 65 | M | 6 |
| CT2 | 3/3 | 0 | 2 | 66 | M | 10 |
| CT3 | 3/3 | 0 | 1 | 70 | M | 4.5 |
| CT4 | 3/3 | 0 | 2 | 69 | M | 6 |
| CT5 | 3/3 | 0 | 1 | 57 | F | 17 |
| CT6 | 3/3 | 0 | 2 | 78 | F | 11.5 |
| CT7 | 3/3 | 0 | 3 | 92 | F | 15.5 |
| CT8 | 3/3 | 0 | 2 | 94 | M | 5.5 |
| CT9 | 3/3 | 1 | 3 | 91 | F | 6 |
| CT10 | 3/3 | 0 | 1 | 70 | M | 2.5 |
| AD1 | 2/3 | 3 | 5 | 73 | M | 4 |
| AD2 | 2/3 | 2 | 4 | 88 | F | 2.5 |
| AD3 | 2/3 | 3 | 4 | 74 | M | 6 |
| AD4 | 2/3 | 3 | 5.5 | 87 | M | 20 |
| AD5 | 2/3 | 3 | 6 | 78 | M | 27 |
| AD6 | 2/3 | 3 | 2 | 85 | F | 9 |
| AD7 | 2/3 | 3 | 6 | 67 | M | 6.5 |
| AD8 | 2/3 | 3 | 6 | 62 | M | 6 |
| AD9 | 3/3 | 3 | 5 | 89 | M | 5 |
| AD10 | 3/3 | 3 | 6 | 66 | M | 5 |
| AD11 | 3/3 | 3 | 5 | 74 | F | 3.5 |
| AD12 | 3/3 | 3 | 6 | 92 | F | 6 |
| AD13 | 3/3 | 3 | 5.5 | 77 | M | 24 |
| AD14 | 3/3 | 3 | 6 | 67 | M | 18 |
| AD15 | 3/3 | 3 | 6 | 74 | M | 2.5 |
| AD16 | 3/3 | 3 | 6 | 75 | M | 15 |
| AD17* | 4/4 | 3 | 6 | 62 | M | 14.5 |
| AD18 | 4/4 | 3 | 5 | 75 | F | 8 |
| AD19 | 4/4 | 3 | 5 | 88 | M | 4.25 |
| AD20 | 4/4 | 3 | 6 | 77 | M | 9 |
| AD21 | 4/4 | 3 | 6 | 88 | M | 9.5 |
| AD22* | 4/4 | 3 | 6 | 83 | M | 5 |
| AD23 | 4/4 | 3 | 6 | 63 | F | 28.5 |
| AD24 | 4/4 | 3 | 6 | 72 | M | 3 |

**Supplementary Table 1**

| Case Group | CERAD | | Braak | Age | Sex (M/F) | PMI (h) |
| --- | --- | --- | --- | --- | --- | --- |
| Control E3/3 | | 0.1±0.3 | 1.8±0.8 | 75.2±12.9 | 6/4 | 8.5±4.9 |
| AD E2/3 | | 2.9±0.4 | 4.8±1.4 | 76.8±9.5 | 6/2 | 10.1±8.7 |
| AD E3/3 | | 3.0±0.0 | 5.7±0.5 | 76.8±9.3 | 6/2 | 9.9±8.0 |
| AD E4/4 | | 3.0±0.0 | 5.8±0.5 | 76.0±10.1 | 6/2 | 10.2±8.2 |

**Supplementary Table 2**

| Cell Type | FET *P* Value |
| --- | --- |
| Endothelia | M32 = 0.077  **M11 = 8.4E-13** |
| Microglia | M3 = 0.077  M2 = 0.078  M4 = 0.0015 |
| Astrocytes | M2 = 6.2E-8  M22 = 0.0002  M11 = 0.029  M4 = 0.038 |
| Neurons | M12 = 0.004  M17 = 0.029  M15 = 0.027  M1 = 0.002 |
| Oligodendrocytes | M3 = 5.6E-6 |

**Supplementary Table 3**

| Case Group | CERAD | | Braak | Age | Sex (M/F) | PMI (h) |
| --- | --- | --- | --- | --- | --- | --- |
| Control E2/3 | | zero/sparse/mod/freq | 3.5±0.8 | 85.3±7.8 | 8/10 | 2.7±0.7 |
| Control E3/3 | | zero/sparse/mod/freq | 3.1±0.9 | 86.9±7.0 | 35/24 | 3.0±0.8 |
| AD E2/3 | | freq | 4.5±0.8 | 88.3±11.1 | 3/3 | 3.1±0.8 |
| AD E3/3 | | mod/freq | 5.1±0.5 | 84.8±7.5 | 14/16 | 3.0±0.9 |
| AD E4/4 | | mod/freq | 5.3±0.5 | 79.9±6.0 | 6/2 | 3.1±0.8 |

**Supplementary Table 4**
